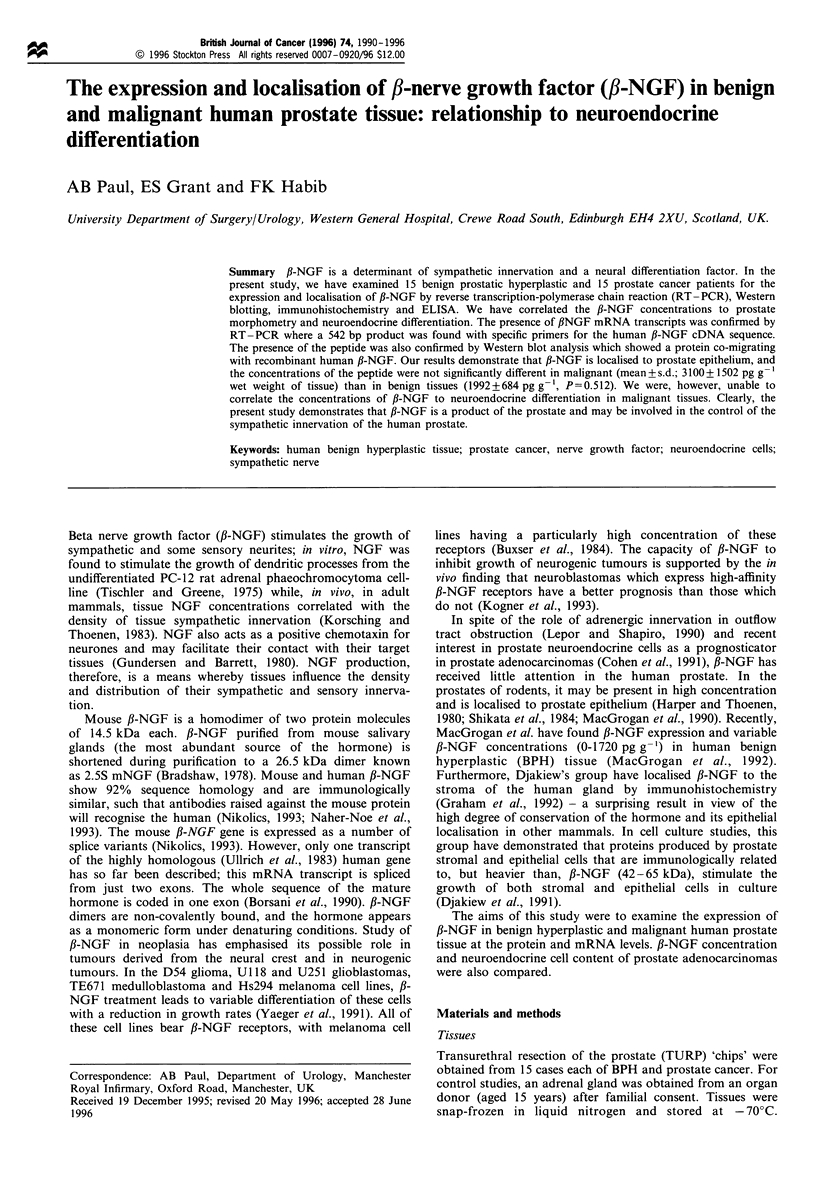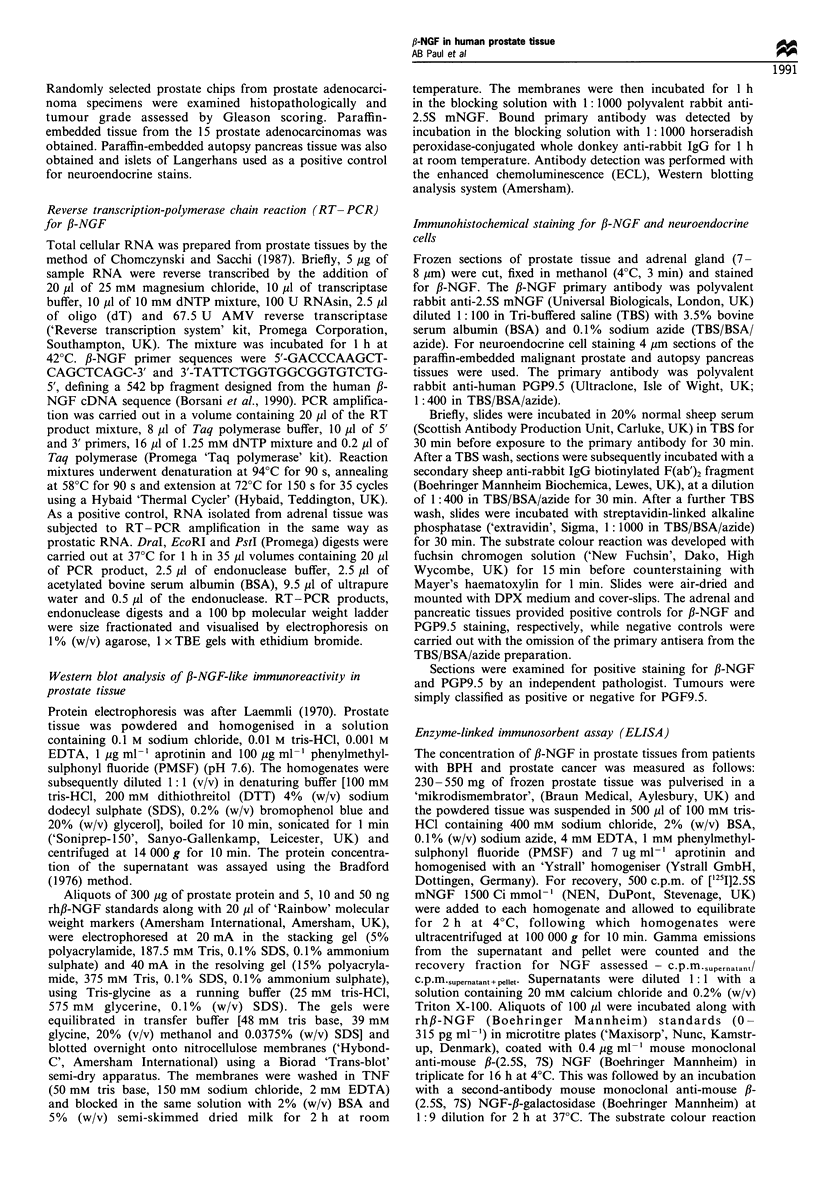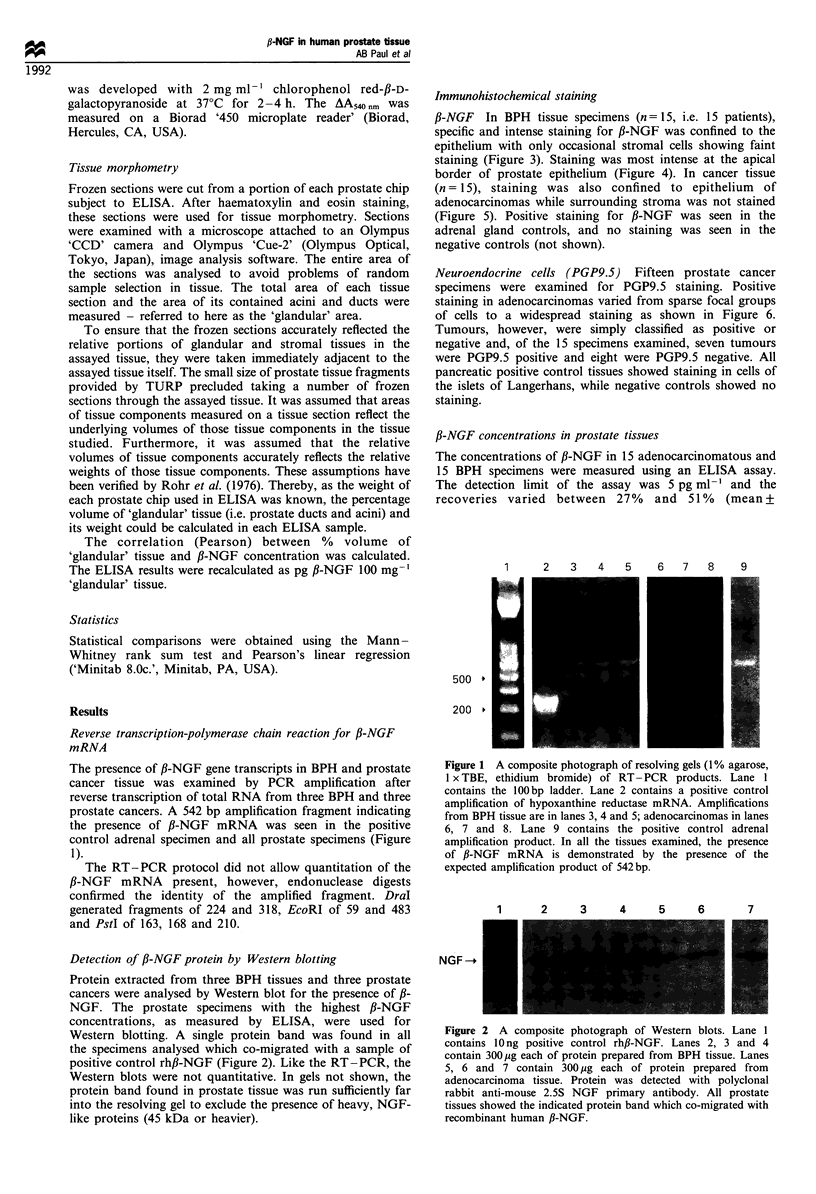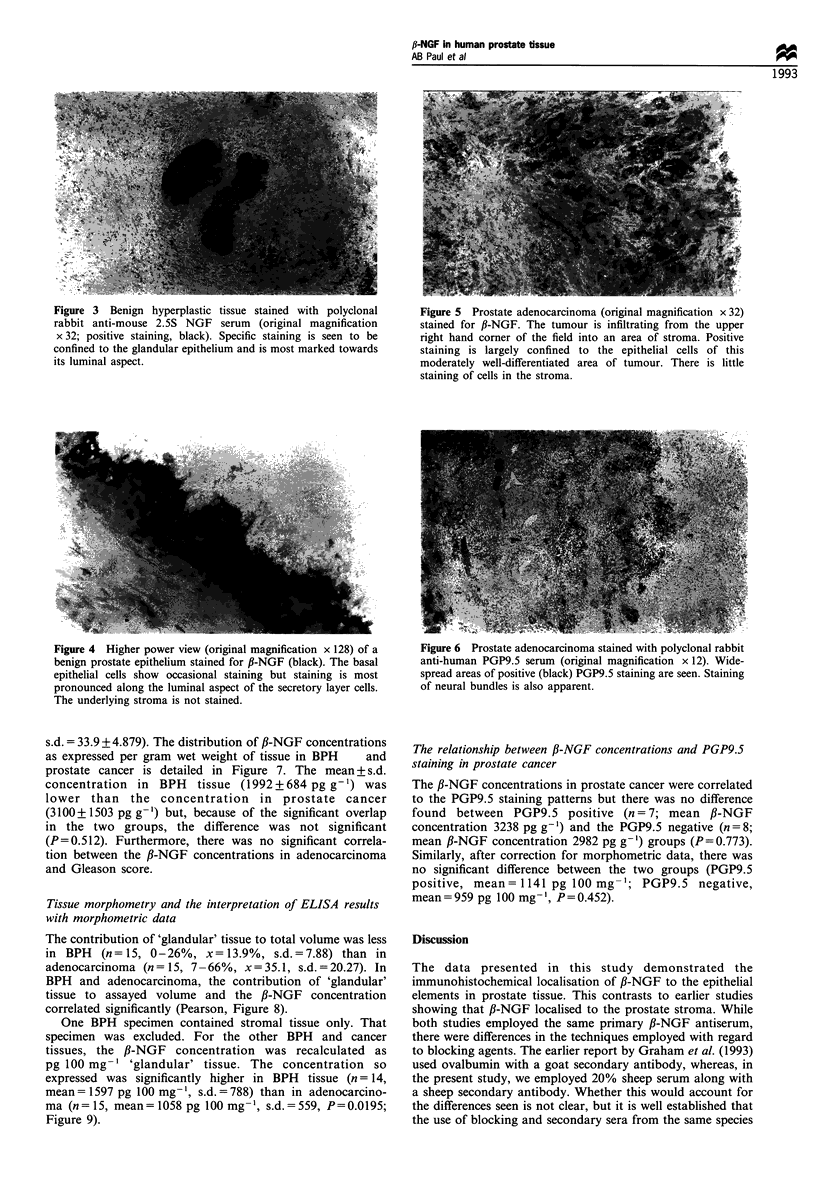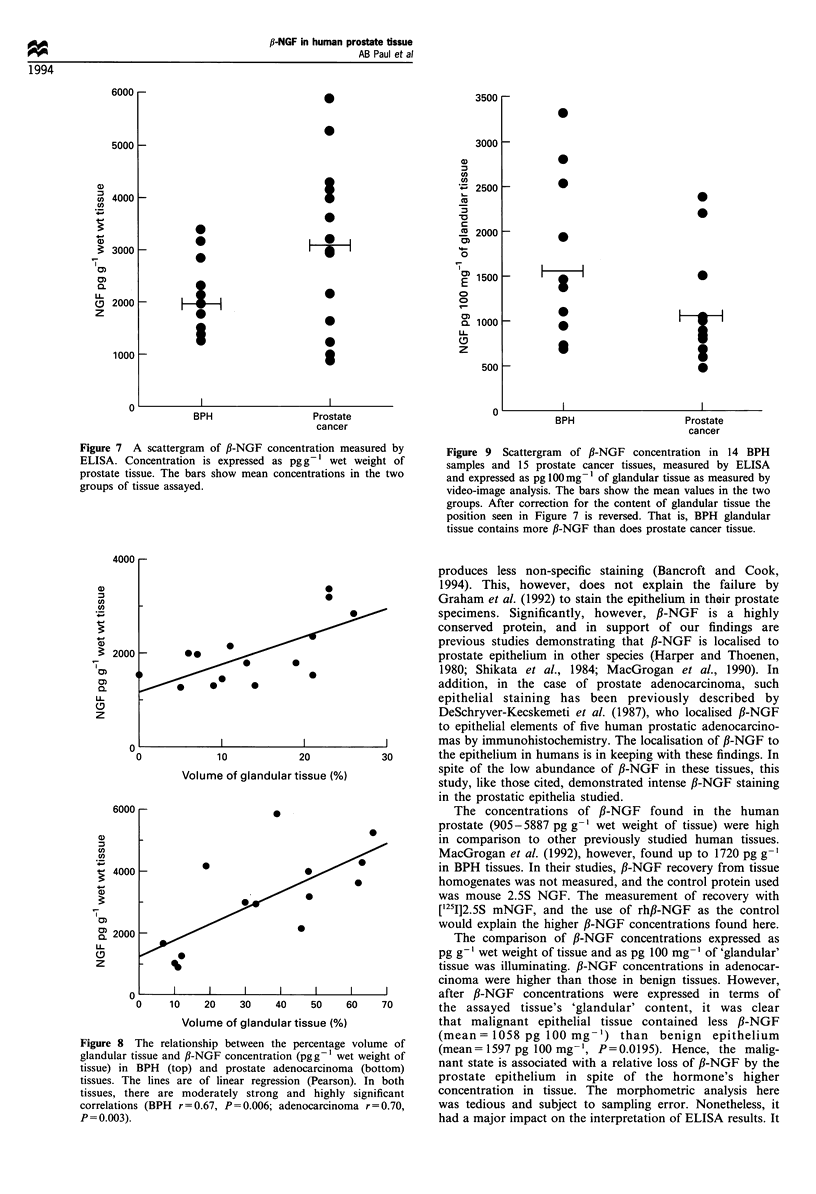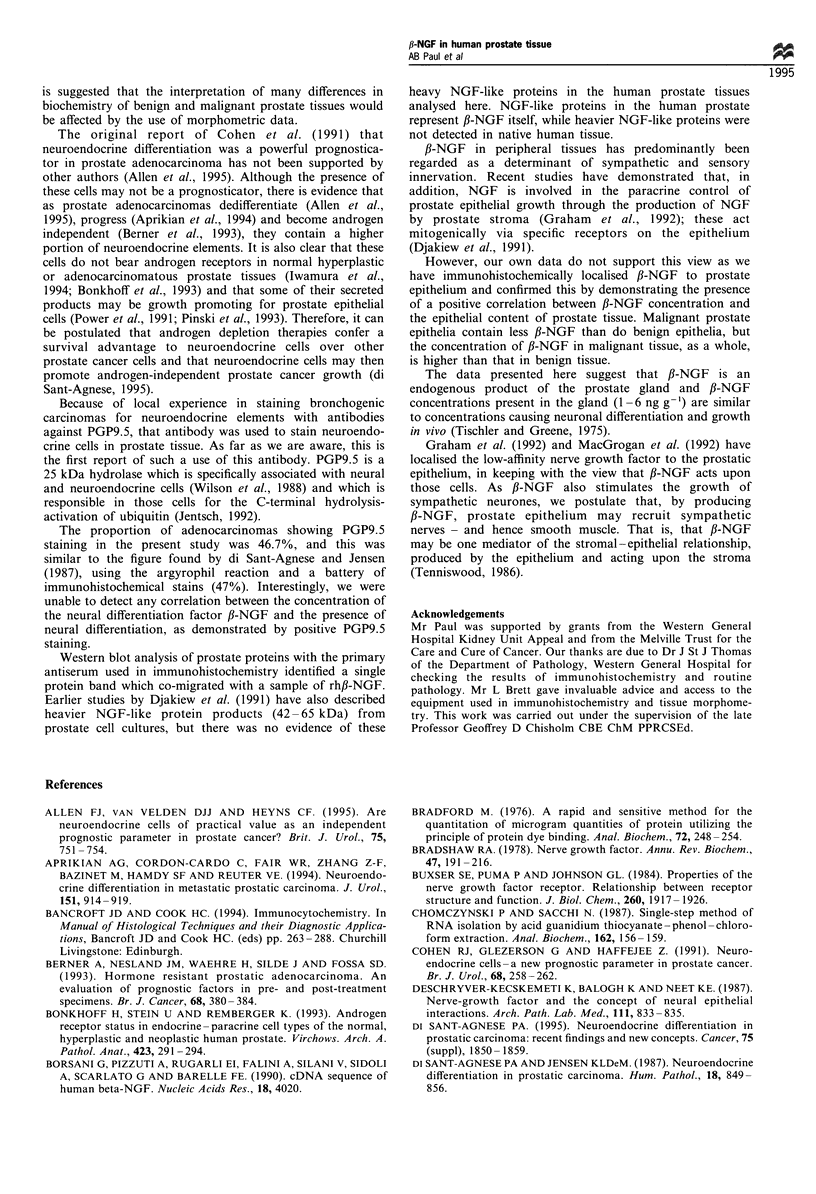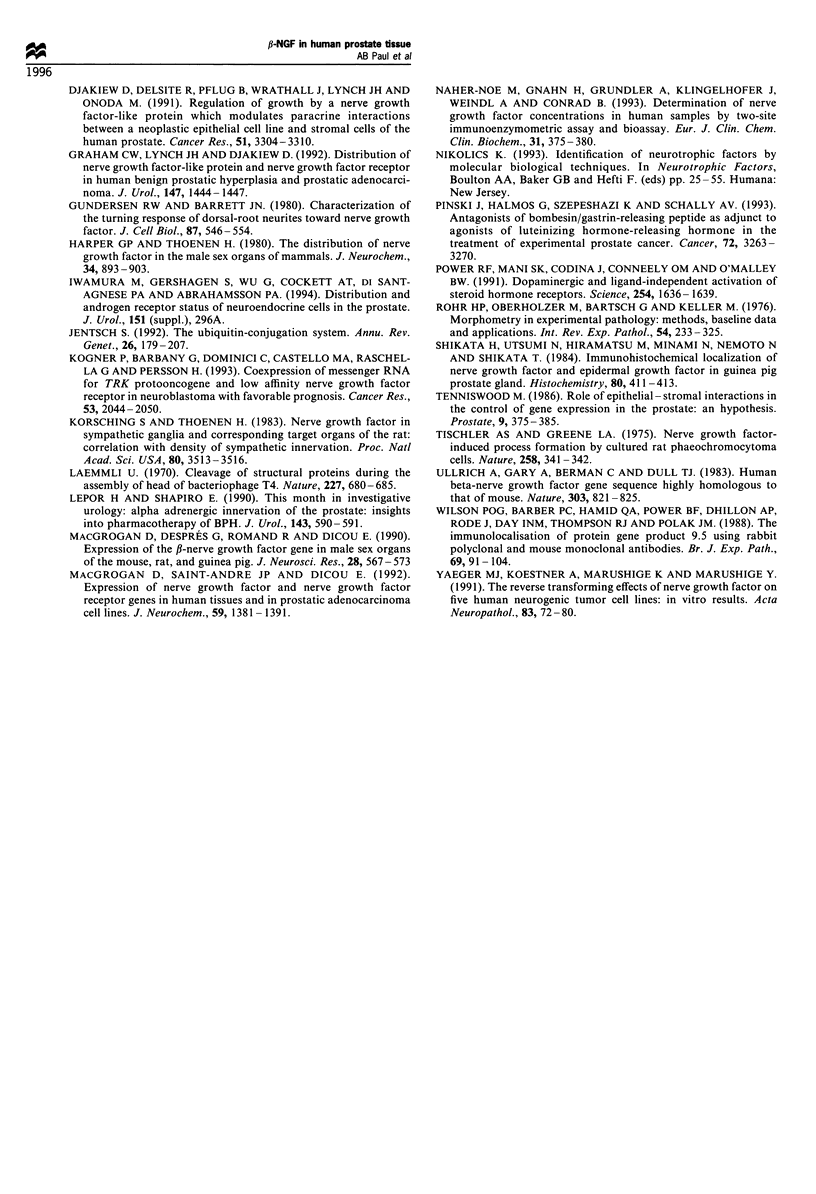# The expression and localisation of beta-nerve growth factor (beta-NGF) in benign and malignant human prostate tissue: relationship to neuroendocrine differentiation.

**DOI:** 10.1038/bjc.1996.665

**Published:** 1996-12

**Authors:** A. B. Paul, E. S. Grant, F. K. Habib

**Affiliations:** University Department of Surgery/Urology, Western General Hospital, Edinburgh, Scotland, UK.

## Abstract

**Images:**


British Journal of Cancer (1996) 74, 1990-1996
© 1996 Stockton Press All rights reserved 0007-0920/96 $12.00

### The expression and localisation of β-nerve growth factor (β-NGF) in benign and malignant human prostate tissue: relationship to neuroendocrine differentiation

AB Paul, ES Grant and FK Habib

*University Department of Surgery/Urology, Western General Hospital, Crewe Road South, Edinburgh EH4 2XU, Scotland, UK.*

**Summary** β-NGF is a determinant of sympathetic innervation and a neural differentiation factor. In the present study, we have examined 15 benign prostatic hyperplastic and 15 prostate cancer patients for the expression and localisation of β-NGF by reverse transcription-polymerase chain reaction (RT-PCR), Western blotting, immunohistochemistry and ELISA. We have correlated the β-NGF concentrations to prostate morphometry and neuroendocrine differentiation. The presence of βNGF mRNA transcripts was confirmed by RT-PCR where a 542 bp product was found with specific primers for the human β-NGF cDNA sequence. The presence of the peptide was also confirmed by Western blot analysis which showed a protein co-migrating with recombinant human β-NGF. Our results demonstrate that β-NGF is localised to prostate epithelium, and the concentrations of the peptide were not significantly different in malignant (mean ± s.d.; 3100 ± 1502 pg $g^{-1}$ wet weight of tissue) than in benign tissues (1992 ± 684 pg $g^{-1}$, $P=0.512$). We were, however, unable to correlate the concentrations of β-NGF to neuroendocrine differentiation in malignant tissues. Clearly, the present study demonstrates that β-NGF is a product of the prostate and may be involved in the control of the sympathetic innervation of the human prostate.

**Keywords:** human benign hyperplastic tissue; prostate cancer, nerve growth factor; neuroendocrine cells; sympathetic nerve

Beta nerve growth factor (β-NGF) stimulates the growth of sympathetic and some sensory neurites; *in vitro*, NGF was found to stimulate the growth of dendritic processes from the undifferentiated PC-12 rat adrenal phaeochromocytoma cell-line (Tischler and Greene, 1975) while, *in vivo*, in adult mammals, tissue NGF concentrations correlated with the density of tissue sympathetic innervation (Korsching and Thoenen, 1983). NGF also acts as a positive chemotaxin for neurones and may facilitate their contact with their target tissues (Gundersen and Barrett, 1980). NGF production, therefore, is a means whereby tissues influence the density and distribution of their sympathetic and sensory innervation.

Mouse β-NGF is a homodimer of two protein molecules of 14.5 kDa each. β-NGF purified from mouse salivary glands (the most abundant source of the hormone) is shortened during purification to a 26.5 kDa dimer known as 2.5S mNGF (Bradshaw, 1978). Mouse and human β-NGF show 92% sequence homology and are immunologically similar, such that antibodies raised against the mouse protein will recognise the human (Nikolics, 1993; Naher-Noe *et al.*, 1993). The mouse *β-NGF* gene is expressed as a number of splice variants (Nikolics, 1993). However, only one transcript of the highly homologous (Ullrich *et al.*, 1983) human gene has so far been described; this mRNA transcript is spliced from just two exons. The whole sequence of the mature hormone is coded in one exon (Borsani *et al.*, 1990). β-NGF dimers are non-covalently bound, and the hormone appears as a monomeric form under denaturing conditions. Study of β-NGF in neoplasia has emphasised its possible role in tumours derived from the neural crest and in neurogenic tumours. In the D54 glioma, U118 and U251 glioblastomas, TE671 medulloblastoma and Hs294 melanoma cell lines, β-NGF treatment leads to variable differentiation of these cells with a reduction in growth rates (Yaeger *et al.*, 1991). All of these cell lines bear β-NGF receptors, with melanoma cell lines having a particularly high concentration of these receptors (Buxser *et al.*, 1984). The capacity of β-NGF to inhibit growth of neurogenic tumours is supported by the *in vivo* finding that neuroblastomas which express high-affinity β-NGF receptors have a better prognosis than those which do not (Kogner *et al.*, 1993).

In spite of the role of adrenergic innervation in outflow tract obstruction (Lepor and Shapiro, 1990) and recent interest in prostate neuroendocrine cells as a prognosticator in prostate adenocarcinomas (Cohen *et al.*, 1991), β-NGF has received little attention in the human prostate. In the prostates of rodents, it may be present in high concentration and is localised to prostate epithelium (Harper and Thoenen, 1980; Shikata *et al.*, 1984; MacGrogan *et al.*, 1990). Recently, MacGrogan *et al.* have found β-NGF expression and variable β-NGF concentrations (0-1720 pg $g^{-1}$) in human benign hyperplastic (BPH) tissue (MacGrogan *et al.*, 1992). Furthermore, Djakiew's group have localised β-NGF to the stroma of the human gland by immunohistochemistry (Graham *et al.*, 1992) – a surprising result in view of the high degree of conservation of the hormone and its epithelial localisation in other mammals. In cell culture studies, this group have demonstrated that proteins produced by prostate stromal and epithelial cells that are immunologically related to, but heavier than, β-NGF (42-65 kDa), stimulate the growth of both stromal and epithelial cells in culture (Djakiew *et al.*, 1991).

The aims of this study were to examine the expression of β-NGF in benign hyperplastic and malignant human prostate tissue at the protein and mRNA levels. β-NGF concentration and neuroendocrine cell content of prostate adenocarcinomas were also compared.

### Materials and methods

### *Tissues*

Transurethral resection of the prostate (TURP) 'chips' were obtained from 15 cases each of BPH and prostate cancer. For control studies, an adrenal gland was obtained from an organ donor (aged 15 years) after familial consent. Tissues were snap-frozen in liquid nitrogen and stored at −70°C.

Correspondence: AB Paul, Department of Urology, Manchester Royal Infirmary, Oxford Road, Manchester, UK
Received 19 December 1995; revised 20 May 1996; accepted 28 June 1996

Randomly selected prostate chips from prostate adenocarcinoma specimens were examined histopathologically and tumour grade assessed by Gleason scoring. Paraffin-embedded tissue from the 15 prostate adenocarcinomas was obtained. Paraffin-embedded autopsy pancreas tissue was also obtained and islets of Langerhans used as a positive control for neuroendocrine stains.

### *Reverse transcription-polymerase chain reaction (RT–PCR) for β-NGF*

Total cellular RNA was prepared from prostate tissues by the method of Chomczynski and Sacchi (1987). Briefly, 5 μg of sample RNA were reverse transcribed by the addition of 20 μl of 25 mM magnesium chloride, 10 μl of transcriptase buffer, 10 μl of 10 mM dNTP mixture, 100 U RNAsin, 2.5 μl of oligo (dT) and 67.5 U AMV reverse transcriptase ('Reverse transcription system' kit, Promega Corporation, Southampton, UK). The mixture was incubated for 1 h at 42°C. β-NGF primer sequences were 5′-GACCCAAGCTCAGCTCAGC-3′ and 3′-TATTCTGGTGGCGGTGTCTG-5′, defining a 542 bp fragment designed from the human β-NGF cDNA sequence (Borsani *et al*., 1990). PCR amplification was carried out in a volume containing 20 μl of the RT product mixture, 8 μl of *Taq* polymerase buffer, 10 μl of 5′ and 3′ primers, 16 μl of 1.25 mM dNTP mixture and 0.2 μl of *Taq* polymerase (Promega 'Taq polymerase' kit). Reaction mixtures underwent denaturation at 94°C for 90 s, annealing at 58°C for 90 s and extension at 72°C for 150 s for 35 cycles using a Hybaid 'Thermal Cycler' (Hybaid, Teddington, UK). As a positive control, RNA isolated from adrenal tissue was subjected to RT–PCR amplification in the same way as prostatic RNA. *Dra*I, *Eco*RI and *Pst*I (Promega) digests were carried out at 37°C for 1 h in 35 μl volumes containing 20 μl of PCR product, 2.5 μl of endonuclease buffer, 2.5 μl of acetylated bovine serum albumin (BSA), 9.5 μl of ultrapure water and 0.5 μl of the endonuclease. RT–PCR products, endonuclease digests and a 100 bp molecular weight ladder were size fractionated and visualised by electrophoresis on 1% (w/v) agarose, 1 × TBE gels with ethidium bromide.

### *Western blot analysis of β-NGF-like immunoreactivity in prostate tissue*

Protein electrophoresis was after Laemmli (1970). Prostate tissue was powdered and homogenised in a solution containing 0.1 M sodium chloride, 0.01 M tris-HCl, 0.001 M EDTA, 1 μg ml$^{-1}$ aprotinin and 100 μg ml$^{-1}$ phenylmethylsulphonyl fluoride (PMSF) (pH 7.6). The homogenates were subsequently diluted 1 : 1 (v/v) in denaturing buffer [100 mM tris-HCl, 200 mM dithiothreitol (DTT) 4% (w/v) sodium dodecyl sulphate (SDS), 0.2% (w/v) bromophenol blue and 20% (w/v) glycerol], boiled for 10 min, sonicated for 1 min ('Soniprep-150', Sanyo-Gallenkamp, Leicester, UK) and centrifuged at 14 000 *g* for 10 min. The protein concentration of the supernatant was assayed using the Bradford (1976) method.

Aliquots of 300 μg of prostate protein and 5, 10 and 50 ng rhβ-NGF standards along with 20 μl of 'Rainbow' molecular weight markers (Amersham International, Amersham, UK), were electrophoresed at 20 mA in the stacking gel (5% polyacrylamide, 187.5 mM Tris, 0.1% SDS, 0.1% ammonium sulphate) and 40 mA in the resolving gel (15% polyacrylamide, 375 mM Tris, 0.1% SDS, 0.1% ammonium sulphate), using Tris-glycine as a running buffer (25 mM tris-HCl, 575 mM glycerine, 0.1% (w/v) SDS). The gels were equilibrated in transfer buffer [48 mM tris base, 39 mM glycine, 20% (v/v) methanol and 0.0375% (w/v) SDS] and blotted overnight onto nitrocellulose membranes ('Hybond-C', Amersham International) using a Biorad 'Trans-blot' semi-dry apparatus. The membranes were washed in TNF (50 mM tris base, 150 mM sodium chloride, 2 mM EDTA) and blocked in the same solution with 2% (w/v) BSA and 5% (w/v) semi-skimmed dried milk for 2 h at room temperature. The membranes were then incubated for 1 h in the blocking solution with 1 : 1000 polyvalent rabbit anti-2.5S mNGF. Bound primary antibody was detected by incubation in the blocking solution with 1 : 1000 horseradish peroxidase-conjugated whole donkey anti-rabbit IgG for 1 h at room temperature. Antibody detection was performed with the enhanced chemoluminescence (ECL), Western blotting analysis system (Amersham).

### *Immunohistochemical staining for β-NGF and neuroendocrine cells*

Frozen sections of prostate tissue and adrenal gland (7–8 μm) were cut, fixed in methanol (4°C, 3 min) and stained for β-NGF. The β-NGF primary antibody was polyvalent rabbit anti-2.5S mNGF (Universal Biologicals, London, UK) diluted 1 : 100 in Tri-buffered saline (TBS) with 3.5% bovine serum albumin (BSA) and 0.1% sodium azide (TBS/BSA/azide). For neuroendocrine cell staining 4 μm sections of the paraffin-embedded malignant prostate and autopsy pancreas tissues were used. The primary antibody was polyvalent rabbit anti-human PGP9.5 (Ultraclone, Isle of Wight, UK; 1 : 400 in TBS/BSA/azide).

Briefly, slides were incubated in 20% normal sheep serum (Scottish Antibody Production Unit, Carluke, UK) in TBS for 30 min before exposure to the primary antibody for 30 min. After a TBS wash, sections were subsequently incubated with a secondary sheep anti-rabbit IgG biotinylated $F(ab')_2$ fragment (Boehringer Mannheim Biochemica, Lewes, UK), at a dilution of 1 : 400 in TBS/BSA/azide for 30 min. After a further TBS wash, slides were incubated with streptavidin-linked alkaline phosphatase ('extravidin', Sigma, 1 : 1000 in TBS/BSA/azide) for 30 min. The substrate colour reaction was developed with fuchsin chromogen solution ('New Fuchsin', Dako, High Wycombe, UK) for 15 min before counterstaining with Mayer's haematoxylin for 1 min. Slides were air-dried and mounted with DPX medium and cover-slips. The adrenal and pancreatic tissues provided positive controls for β-NGF and PGP9.5 staining, respectively, while negative controls were carried out with the omission of the primary antisera from the TBS/BSA/azide preparation.

Sections were examined for positive staining for β-NGF and PGP9.5 by an independent pathologist. Tumours were simply classified as positive or negative for PGF9.5.

### *Enzyme-linked immunosorbent assay (ELISA)*

The concentration of β-NGF in prostate tissues from patients with BPH and prostate cancer was measured as follows: 230–550 mg of frozen prostate tissue was pulverised in a 'mikrodismembrator', (Braun Medical, Aylesbury, UK) and the powdered tissue was suspended in 500 μl of 100 mM tris-HCl containing 400 mM sodium chloride, 2% (w/v) BSA, 0.1% (w/v) sodium azide, 4 mM EDTA, 1 mM phenylmethylsulphonyl fluoride (PMSF) and 7 ug ml$^{-1}$ aprotinin and homogenised with an 'Ystrall' homogeniser (Ystrall GmbH, Dottingen, Germany). For recovery, 500 c.p.m. of [$^{125}$I]2.5S mNGF 1500 Ci mmol$^{-1}$ (NEN, DuPont, Stevenage, UK) were added to each homogenate and allowed to equilibrate for 2 h at 4°C, following which homogenates were ultracentrifuged at 100 000 *g* for 10 min. Gamma emissions from the supernatant and pellet were counted and the recovery fraction for NGF assessed – $\text{c.p.m.}_{\text{supernatant}}/\text{c.p.m.}_{\text{supernatant+pellet}}$. Supernatants were diluted 1 : 1 with a solution containing 20 mM calcium chloride and 0.2% (w/v) Triton X-100. Aliquots of 100 μl were incubated along with rhβ-NGF (Boehringer Mannheim) standards (0–315 pg ml$^{-1}$) in microtitre plates ('Maxisorp', Nunc, Kamstrup, Denmark), coated with 0.4 μg ml$^{-1}$ mouse monoclonal anti-mouse β-(2.5S, 7S) NGF (Boehringer Mannheim) in triplicate for 16 h at 4°C. This was followed by an incubation with a second-antibody mouse monoclonal anti-mouse β-(2.5S, 7S) NGF-β-galactosidase (Boehringer Mannheim) at 1 : 9 dilution for 2 h at 37°C. The substrate colour reaction was developed with 2 mg ml$^{-1}$ chlorophenol red-β-D-galactopyranoside at 37°C for 2–4 h. The $\Delta A_{540\,nm}$ was measured on a Biorad '450 microplate reader' (Biorad, Hercules, CA, USA).

*Tissue morphometry*

Frozen sections were cut from a portion of each prostate chip subject to ELISA. After haematoxylin and eosin staining, these sections were used for tissue morphometry. Sections were examined with a microscope attached to an Olympus 'CCD' camera and Olympus 'Cue-2' (Olympus Optical, Tokyo, Japan), image analysis software. The entire area of the sections was analysed to avoid problems of random sample selection in tissue. The total area of each tissue section and the area of its contained acini and ducts were measured – referred to here as the 'glandular' area.

To ensure that the frozen sections accurately reflected the relative portions of glandular and stromal tissues in the assayed tissue, they were taken immediately adjacent to the assayed tissue itself. The small size of prostate tissue fragments provided by TURP precluded taking a number of frozen sections through the assayed tissue. It was assumed that areas of tissue components measured on a tissue section reflect the underlying volumes of those tissue components in the tissue studied. Furthermore, it was assumed that the relative volumes of tissue components accurately reflects the relative weights of those tissue components. These assumptions have been verified by Rohr *et al.* (1976). Thereby, as the weight of each prostate chip used in ELISA was known, the percentage volume of 'glandular' tissue (i.e. prostate ducts and acini) and its weight could be calculated in each ELISA sample.

The correlation (Pearson) between % volume of 'glandular' tissue and β-NGF concentration was calculated. The ELISA results were recalculated as pg β-NGF 100 mg$^{-1}$ 'glandular' tissue.

*Statistics*

Statistical comparisons were obtained using the Mann–Whitney rank sum test and Pearson's linear regression ('Minitab 8.0c.', Minitab, PA, USA).

### Results

*Reverse transcription-polymerase chain reaction for β-NGF mRNA*

The presence of β-NGF gene transcripts in BPH and prostate cancer tissue was examined by PCR amplification after reverse transcription of total RNA from three BPH and three prostate cancers. A 542 bp amplification fragment indicating the presence of β-NGF mRNA was seen in the positive control adrenal specimen and all prostate specimens (Figure 1).

The RT–PCR protocol did not allow quantitation of the β-NGF mRNA present, however, endonuclease digests confirmed the identity of the amplified fragment. *Dra*I generated fragments of 224 and 318, *Eco*RI of 59 and 483 and *Pst*I of 163, 168 and 210.

*Detection of β-NGF protein by Western blotting*

Protein extracted from three BPH tissues and three prostate cancers were analysed by Western blot for the presence of β-NGF. The prostate specimens with the highest β-NGF concentrations, as measured by ELISA, were used for Western blotting. A single protein band was found in all the specimens analysed which co-migrated with a sample of positive control rhβ-NGF (Figure 2). Like the RT–PCR, the Western blots were not quantitative. In gels not shown, the protein band found in prostate tissue was run sufficiently far into the resolving gel to exclude the presence of heavy, NGF-like proteins (45 kDa or heavier).

*Immunohistochemical staining*

*β-NGF* In BPH tissue specimens ($n$ = 15, i.e. 15 patients), specific and intense staining for β-NGF was confined to the epithelium with only occasional stromal cells showing faint staining (Figure 3). Staining was most intense at the apical border of prostate epithelium (Figure 4). In cancer tissue ($n$ = 15), staining was also confined to epithelium of adenocarcinomas while surrounding stroma was not stained (Figure 5). Positive staining for β-NGF was seen in the adrenal gland controls, and no staining was seen in the negative controls (not shown).

*Neuroendocrine cells (PGP9.5)* Fifteen prostate cancer specimens were examined for PGP9.5 staining. Positive staining in adenocarcinomas varied from sparse focal groups of cells to a widespread staining as shown in Figure 6. Tumours, however, were simply classified as positive or negative and, of the 15 specimens examined, seven tumours were PGP9.5 positive and eight were PGP9.5 negative. All pancreatic positive control tissues showed staining in cells of the islets of Langerhans, while negative controls showed no staining.

*β-NGF concentrations in prostate tissues*

The concentrations of β-NGF in 15 adenocarcinomatous and 15 BPH specimens were measured using an ELISA assay. The detection limit of the assay was 5 pg ml$^{-1}$ and the recoveries varied between 27% and 51% (mean ±

**Figure 1** A composite photograph of resolving gels (1% agarose, 1 × TBE, ethidium bromide) of RT–PCR products. Lane 1 contains the 100 bp ladder. Lane 2 contains a positive control amplification of hypoxanthine reductase mRNA. Amplifications from BPH tissue are in lanes 3, 4 and 5; adenocarcinomas in lanes 6, 7 and 8. Lane 9 contains the positive control adrenal amplification product. In all the tissues examined, the presence of β-NGF mRNA is demonstrated by the presence of the expected amplification product of 542 bp.

**Figure 2** A composite photograph of Western blots. Lane 1 contains 10 ng positive control rhβ-NGF. Lanes 2, 3 and 4 contain 300 μg each of protein prepared from BPH tissue. Lanes 5, 6 and 7 contain 300 μg each of protein prepared from adenocarcinoma tissue. Protein was detected with polyclonal rabbit anti-mouse 2.5S NGF primary antibody. All prostate tissues showed the indicated protein band which co-migrated with recombinant human β-NGF.

**Figure 3** Benign hyperplastic tissue stained with polyclonal rabbit anti-mouse 2.5S NGF serum (original magnification ×32; positive staining, black). Specific staining is seen to be confined to the glandular epithelium and is most marked towards its luminal aspect.

**Figure 5** Prostate adenocarcinoma (original magnification ×32) stained for β-NGF. The tumour is infiltrating from the upper right hand corner of the field into an area of stroma. Positive staining is largely confined to the epithelial cells of this moderately well-differentiated area of tumour. There is little staining of cells in the stroma.

**Figure 4** Higher power view (original magnification ×128) of a benign prostate epithelium stained for β-NGF (black). The basal epithelial cells show occasional staining but staining is most pronounced along the luminal aspect of the secretory layer cells. The underlying stroma is not stained.

**Figure 6** Prostate adenocarcinoma stained with polyclonal rabbit anti-human PGP9.5 serum (original magnification ×12). Widespread areas of positive (black) PGP9.5 staining are seen. Staining of neural bundles is also apparent.

s.d. = 33.9 ± 4.879). The distribution of β-NGF concentrations as expressed per gram wet weight of tissue in BPH and prostate cancer is detailed in Figure 7. The mean ± s.d. concentration in BPH tissue (1992 ± 684 pg $g^{-1}$) was lower than the concentration in prostate cancer (3100 ± 1503 pg $g^{-1}$) but, because of the significant overlap in the two groups, the difference was not significant ($P = 0.512$). Furthermore, there was no significant correlation between the β-NGF concentrations in adenocarcinoma and Gleason score.

*Tissue morphometry and the interpretation of ELISA results with morphometric data*

The contribution of 'glandular' tissue to total volume was less in BPH ($n = 15$, 0–26%, $x = 13.9$%, s.d. = 7.88) than in adenocarcinoma ($n = 15$, 7–66%, $x = 35.1$, s.d. = 20.27). In BPH and adenocarcinoma, the contribution of 'glandular' tissue to assayed volume and the β-NGF concentration correlated significantly (Pearson, Figure 8).

One BPH specimen contained stromal tissue only. That specimen was excluded. For the other BPH and cancer tissues, the β-NGF concentration was recalculated as pg 100 $mg^{-1}$ 'glandular' tissue. The concentration so expressed was significantly higher in BPH tissue ($n = 14$, mean = 1597 pg 100 $mg^{-1}$, s.d. = 788) than in adenocarcinoma ($n = 15$, mean = 1058 pg 100 $mg^{-1}$, s.d. = 559, $P = 0.0195$; Figure 9).

*The relationship between β-NGF concentrations and PGP9.5 staining in prostate cancer*

The β-NGF concentrations in prostate cancer were correlated to the PGP9.5 staining patterns but there was no difference found between PGP9.5 positive ($n = 7$; mean β-NGF concentration 3238 pg $g^{-1}$) and the PGP9.5 negative ($n = 8$; mean β-NGF concentration 2982 pg $g^{-1}$) groups ($P = 0.773$). Similarly, after correction for morphometric data, there was no significant difference between the two groups (PGP9.5 positive, mean = 1141 pg 100 $mg^{-1}$; PGP9.5 negative, mean = 959 pg 100 $mg^{-1}$, $P = 0.452$).

### Discussion

The data presented in this study demonstrated the immunohistochemical localisation of β-NGF to the epithelial elements in prostate tissue. This contrasts to earlier studies showing that β-NGF localised to the prostate stroma. While both studies employed the same primary β-NGF antiserum, there were differences in the techniques employed with regard to blocking agents. The earlier report by Graham *et al.* (1993) used ovalbumin with a goat secondary antibody, whereas, in the present study, we employed 20% sheep serum along with a sheep secondary antibody. Whether this would account for the differences seen is not clear, but it is well established that the use of blocking and secondary sera from the same species

**Figure 7** A scattergram of β-NGF concentration measured by ELISA. Concentration is expressed as pg g$^{-1}$ wet weight of prostate tissue. The bars show mean concentrations in the two groups of tissue assayed.

**Figure 8** The relationship between the percentage volume of glandular tissue and β-NGF concentration (pg g$^{-1}$ wet weight of tissue) in BPH (top) and prostate adenocarcinoma (bottom) tissues. The lines are of linear regression (Pearson). In both tissues, there are moderately strong and highly significant correlations (BPH $r = 0.67$, $P = 0.006$; adenocarcinoma $r = 0.70$, $P = 0.003$).

**Figure 9** Scattergram of β-NGF concentration in 14 BPH samples and 15 prostate cancer tissues, measured by ELISA and expressed as pg 100 mg$^{-1}$ of glandular tissue as measured by video-image analysis. The bars show the mean values in the two groups. After correction for the content of glandular tissue the position seen in Figure 7 is reversed. That is, BPH glandular tissue contains more β-NGF than does prostate cancer tissue.

produces less non-specific staining (Bancroft and Cook, 1994). This, however, does not explain the failure by Graham *et al.* (1992) to stain the epithelium in their prostate specimens. Significantly, however, β-NGF is a highly conserved protein, and in support of our findings are previous studies demonstrating that β-NGF is localised to prostate epithelium in other species (Harper and Thoenen, 1980; Shikata *et al.*, 1984; MacGrogan *et al.*, 1990). In addition, in the case of prostate adenocarcinoma, such epithelial staining has been previously described by DeSchryver-Kecskemeti *et al.* (1987), who localised β-NGF to epithelial elements of five human prostatic adenocarcinomas by immunohistochemistry. The localisation of β-NGF to the epithelium in humans is in keeping with these findings. In spite of the low abundance of β-NGF in these tissues, this study, like those cited, demonstrated intense β-NGF staining in the prostatic epithelia studied.

The concentrations of β-NGF found in the human prostate (905–5887 pg g$^{-1}$ wet weight of tissue) were high in comparison to other previously studied human tissues. MacGrogan *et al.* (1992), however, found up to 1720 pg g$^{-1}$ in BPH tissues. In their studies, β-NGF recovery from tissue homogenates was not measured, and the control protein used was mouse 2.5S NGF. The measurement of recovery with [$^{125}$I]2.5S mNGF, and the use of rhβ-NGF as the control would explain the higher β-NGF concentrations found here.

The comparison of β-NGF concentrations expressed as pg g$^{-1}$ wet weight of tissue and as pg 100 mg$^{-1}$ of 'glandular' tissue was illuminating. β-NGF concentrations in adenocarcinoma were higher than those in benign tissues. However, after β-NGF concentrations were expressed in terms of the assayed tissue's 'glandular' content, it was clear that malignant epithelial tissue contained less β-NGF (mean = 1058 pg 100 mg$^{-1}$) than benign epithelium (mean = 1597 pg 100 mg$^{-1}$, $P = 0.0195$). Hence, the malignant state is associated with a relative loss of β-NGF by the prostate epithelium in spite of the hormone's higher concentration in tissue. The morphometric analysis here was tedious and subject to sampling error. Nonetheless, it had a major impact on the interpretation of ELISA results. It is suggested that the interpretation of many differences in biochemistry of benign and malignant prostate tissues would be affected by the use of morphometric data.

The original report of Cohen *et al.* (1991) that neuroendocrine differentiation was a powerful prognosticator in prostate adenocarcinoma has not been supported by other authors (Allen *et al.*, 1995). Although the presence of these cells may not be a prognosticator, there is evidence that as prostate adenocarcinomas dedifferentiate (Allen *et al.*, 1995), progress (Aprikian *et al.*, 1994) and become androgen independent (Berner *et al.*, 1993), they contain a higher portion of neuroendocrine elements. It is also clear that these cells do not bear androgen receptors in normal hyperplastic or adenocarcinomatous prostate tissues (Iwamura *et al.*, 1994; Bonkhoff *et al.*, 1993) and that some of their secreted products may be growth promoting for prostate epithelial cells (Power *et al.*, 1991; Pinski *et al.*, 1993). Therefore, it can be postulated that androgen depletion therapies confer a survival advantage to neuroendocrine cells over other prostate cancer cells and that neuroendocrine cells may then promote androgen-independent prostate cancer growth (di Sant-Agnese, 1995).

Because of local experience in staining bronchogenic carcinomas for neuroendocrine elements with antibodies against PGP9.5, that antibody was used to stain neuroendocrine cells in prostate tissue. As far as we are aware, this is the first report of such a use of this antibody. PGP9.5 is a 25 kDa hydrolase which is specifically associated with neural and neuroendocrine cells (Wilson *et al.*, 1988) and which is responsible in those cells for the C-terminal hydrolysis-activation of ubiquitin (Jentsch, 1992).

The proportion of adenocarcinomas showing PGP9.5 staining in the present study was 46.7%, and this was similar to the figure found by di Sant-Agnese and Jensen (1987), using the argyrophil reaction and a battery of immunohistochemical stains (47%). Interestingly, we were unable to detect any correlation between the concentration of the neural differentiation factor β-NGF and the presence of neural differentiation, as demonstrated by positive PGP9.5 staining.

Western blot analysis of prostate proteins with the primary antiserum used in immunohistochemistry identified a single protein band which co-migrated with a sample of rhβ-NGF. Earlier studies by Djakiew *et al.* (1991) have also described heavier NGF-like protein products (42–65 kDa) from prostate cell cultures, but there was no evidence of these heavy NGF-like proteins in the human prostate tissues analysed here. NGF-like proteins in the human prostate represent β-NGF itself, while heavier NGF-like proteins were not detected in native human tissue.

β-NGF in peripheral tissues has predominantly been regarded as a determinant of sympathetic and sensory innervation. Recent studies have demonstrated that, in addition, NGF is involved in the paracrine control of prostate epithelial growth through the production of NGF by prostate stroma (Graham *et al.*, 1992); these act mitogenically via specific receptors on the epithelium (Djakiew *et al.*, 1991).

However, our own data do not support this view as we have immunohistochemically localised β-NGF to prostate epithelium and confirmed this by demonstrating the presence of a positive correlation between β-NGF concentration and the epithelial content of prostate tissue. Malignant prostate epithelia contain less β-NGF than do benign epithelia, but the concentration of β-NGF in malignant tissue, as a whole, is higher than that in benign tissue.

The data presented here suggest that β-NGF is an endogenous product of the prostate gland and β-NGF concentrations present in the gland (1–6 ng $g^{-1}$) are similar to concentrations causing neuronal differentiation and growth *in vivo* (Tischler and Greene, 1975).

Graham *et al.* (1992) and MacGrogan *et al.* (1992) have localised the low-affinity nerve growth factor to the prostatic epithelium, in keeping with the view that β-NGF acts upon those cells. As β-NGF also stimulates the growth of sympathetic neurones, we postulate that, by producing β-NGF, prostate epithelium may recruit sympathetic nerves – and hence smooth muscle. That is, that β-NGF may be one mediator of the stromal–epithelial relationship, produced by the epithelium and acting upon the stroma (Tenniswood, 1986).

**Acknowledgements**

Mr Paul was supported by grants from the Western General Hospital Kidney Unit Appeal and from the Melville Trust for the Care and Cure of Cancer. Our thanks are due to Dr J St J Thomas of the Department of Pathology, Western General Hospital for checking the results of immunohistochemistry and routine pathology. Mr L Brett gave invaluable advice and access to the equipment used in immunohistochemistry and tissue morphometry. This work was carried out under the supervision of the late Professor Geoffrey D Chisholm CBE ChM PPRCSEd.

### References

ALLEN FJ, VAN VELDEN DJJ AND HEYNS CF. (1995). Are neuroendocrine cells of practical value as an independent prognostic parameter in prostate cancer? *Brit. J. Urol.*, **75**, 751–754.

APRIKIAN AG, CORDON-CARDO C, FAIR WR, ZHANG Z-F, BAZINET M, HAMDY SF AND REUTER VE. (1994). Neuroendocrine differentiation in metastatic prostatic carcinoma. *J. Urol.*, **151**, 914–919.

BANCROFT JD AND COOK HC. (1994). Immunocytochemistry. In *Manual of Histological Techniques and their Diagnostic Applications*, Bancroft JD and Cook HC. (eds) pp. 263–288. Churchill Livingstone: Edinburgh.

BERNER A, NESLAND JM, WAEHRE H, SILDE J AND FOSSA SD. (1993). Hormone resistant prostatic adenocarcinoma. An evaluation of prognostic factors in pre- and post-treatment specimens. *Br. J. Cancer*, **68**, 380–384.

BONKHOFF H, STEIN U AND REMBERGER K. (1993). Androgen receptor status in endocrine–paracrine cell types of the normal, hyperplastic and neoplastic human prostate. *Virchows. Arch. A. Pathol. Anat.*, **423**, 291–294.

BORSANI G, PIZZUTI A, RUGARLI EI, FALINI A, SILANI V, SIDOLI A, SCARLATO G AND BARELLE FE. (1990). cDNA sequence of human beta-NGF. *Nucleic Acids Res.*, **18**, 4020.

BRADFORD M. (1976). A rapid and sensitive method for the quantitation of microgram quantities of protein utilizing the principle of protein dye binding. *Anal. Biochem.*, **72**, 248–254.

BRADSHAW RA. (1978). Nerve growth factor. *Annu. Rev. Biochem.*, **47**, 191–216.

BUXSER SE, PUMA P AND JOHNSON GL. (1984). Properties of the nerve growth factor receptor. Relationship between receptor structure and function. *J. Biol. Chem.*, **260**, 1917–1926.

CHOMCZYNSKI P AND SACCHI N. (1987). Single-step method of RNA isolation by acid guanidium thiocyanate–phenol–chloroform extraction. *Anal. Biochem.*, **162**, 156–159.

COHEN RJ, GLEZERSON G AND HAFFEJEE Z. (1991). Neuroendocrine cells–a new prognostic parameter in prostate cancer. *Br. J. Urol.*, **68**, 258–262.

DESCHRYVER-KECSKEMETI K, BALOGH K AND NEET KE. (1987). Nerve-growth factor and the concept of neural epithelial interactions. *Arch. Path. Lab. Med.*, **111**, 833–835.

DI SANT-AGNESE PA. (1995). Neuroendocrine differentiation in prostatic carcinoma: recent findings and new concepts. *Cancer*, **75** (suppl), 1850–1859.

DI SANT-AGNESE PA AND JENSEN KLDeM. (1987). Neuroendocrine differentiation in prostatic carcinoma. *Hum. Pathol.*, **18**, 849–856.

DJAKIEW D, DELSITE R, PFLUG B, WRATHALL J, LYNCH JH AND ONODA M. (1991). Regulation of growth by a nerve growth factor-like protein which modulates paracrine interactions between a neoplastic epithelial cell line and stromal cells of the human prostate. *Cancer Res.*, **51,** 3304–3310.

GRAHAM CW, LYNCH JH AND DJAKIEW D. (1992). Distribution of nerve growth factor-like protein and nerve growth factor receptor in human benign prostatic hyperplasia and prostatic adenocarcinoma. *J. Urol.*, **147,** 1444–1447.

GUNDERSEN RW AND BARRETT JN. (1980). Characterization of the turning response of dorsal-root neurites toward nerve growth factor. *J. Cell Biol.*, **87,** 546–554.

HARPER GP AND THOENEN H. (1980). The distribution of nerve growth factor in the male sex organs of mammals. *J. Neurochem.*, **34,** 893–903.

IWAMURA M, GERSHAGEN S, WU G, COCKETT AT, DI SANT-AGNESE PA AND ABRAHAMSSON PA. (1994). Distribution and androgen receptor status of neuroendocrine cells in the prostate. *J. Urol.*, **151** (suppl.), 296A.

JENTSCH S. (1992). The ubiquitin-conjugation system. *Annu. Rev. Genet.*, **26,** 179–207.

KOGNER P, BARBANY G, DOMINICI C, CASTELLO MA, RASCHELLA G AND PERSSON H. (1993). Coexpression of messenger RNA for *TRK* protooncogene and low affinity nerve growth factor receptor in neuroblastoma with favorable prognosis. *Cancer Res.*, **53,** 2044–2050.

KORSCHING S AND THOENEN H. (1983). Nerve growth factor in sympathetic ganglia and corresponding target organs of the rat: correlation with density of sympathetic innervation. *Proc. Natl Acad. Sci. USA*, **80,** 3513–3516.

LAEMMLI U. (1970). Cleavage of structural proteins during the assembly of head of bacteriophage T4. *Nature*, **227,** 680–685.

LEPOR H AND SHAPIRO E. (1990). This month in investigative urology: alpha adrenergic innervation of the prostate: insights into pharmacotherapy of BPH. *J. Urol.*, **143,** 590–591.

MACGROGAN D, DESPRÉS G, ROMAND R AND DICOU E. (1990). Expression of the β-nerve growth factor gene in male sex organs of the mouse, rat, and guinea pig. *J. Neurosci. Res.*, **28,** 567–573

MACGROGAN D, SAINT-ANDRE JP AND DICOU E. (1992). Expression of nerve growth factor and nerve growth factor receptor genes in human tissues and in prostatic adenocarcinoma cell lines. *J. Neurochem.*, **59,** 1381–1391.

NAHER-NOE M, GNAHN H, GRUNDLER A, KLINGELHOFER J, WEINDL A AND CONRAD B. (1993). Determination of nerve growth factor concentrations in human samples by two-site immunoenzymometric assay and bioassay. *Eur. J. Clin. Chem. Clin. Biochem.*, **31,** 375–380.

NIKOLICS K. (1993). Identification of neurotrophic factors by molecular biological techniques. In *Neurotrophic Factors*, Boulton AA, Baker GB and Hefti F. (eds) pp. 25–55. Humana: New Jersey.

PINSKI J, HALMOS G, SZEPESHAZI K AND SCHALLY AV. (1993). Antagonists of bombesin/gastrin-releasing peptide as adjunct to agonists of luteinizing hormone-releasing hormone in the treatment of experimental prostate cancer. *Cancer*, **72,** 3263–3270.

POWER RF, MANI SK, CODINA J, CONNEELY OM AND O'MALLEY BW. (1991). Dopaminergic and ligand-independent activation of steroid hormone receptors. *Science*, **254,** 1636–1639.

ROHR HP, OBERHOLZER M, BARTSCH G AND KELLER M. (1976). Morphometry in experimental pathology: methods, baseline data and applications. *Int. Rev. Exp. Pathol.*, **54,** 233–325.

SHIKATA H, UTSUMI N, HIRAMATSU M, MINAMI N, NEMOTO N AND SHIKATA T. (1984). Immunohistochemical localization of nerve growth factor and epidermal growth factor in guinea pig prostate gland. *Histochemistry*, **80,** 411–413.

TENNISWOOD M. (1986). Role of epithelial–stromal interactions in the control of gene expression in the prostate: an hypothesis. *Prostate*, **9,** 375–385.

TISCHLER AS AND GREENE LA. (1975). Nerve growth factor-induced process formation by cultured rat phaeochromocytoma cells. *Nature*, **258,** 341–342.

ULLRICH A, GARY A, BERMAN C AND DULL TJ. (1983). Human beta-nerve growth factor gene sequence highly homologous to that of mouse. *Nature*, **303,** 821–825.

WILSON POG, BARBER PC, HAMID QA, POWER BF, DHILLON AP, RODE J, DAY INM, THOMPSON RJ AND POLAK JM. (1988). The immunolocalisation of protein gene product 9.5 using rabbit polyclonal and mouse monoclonal antibodies. *Br. J. Exp. Path.*, **69,** 91–104.

YAEGER MJ, KOESTNER A, MARUSHIGE K AND MARUSHIGE Y. (1991). The reverse transforming effects of nerve growth factor on five human neurogenic tumor cell lines: in vitro results. *Acta Neuropathol.*, **83,** 72–80.